# Predicting the Effects of Woody Encroachment on Mammal Communities, Grazing Biomass and Fire Frequency in African Savannas

**DOI:** 10.1371/journal.pone.0137857

**Published:** 2015-09-17

**Authors:** Izak P. J. Smit, Herbert H. T. Prins

**Affiliations:** 1 Scientific Services, South African National Parks, Skukuza, South Africa; 2 Centre for African Ecology, School of Animal, Plant and Environmental Sciences, University of the Witwatersrand, Wits, South Africa; 3 Resource Ecology Group, Wageningen UR, Wageningen, The Netherlands; University of Sydney, AUSTRALIA

## Abstract

With grasslands and savannas covering 20% of the world’s land surface, accounting for 30–35% of worldwide Net Primary Productivity and supporting hundreds of millions of people, predicting changes in tree/grass systems is priority. Inappropriate land management and rising atmospheric CO_2_ levels result in increased woody cover in savannas. Although woody encroachment occurs world-wide, Africa’s tourism and livestock grazing industries may be particularly vulnerable. Forecasts of responses of African wildlife and available grazing biomass to increases in woody cover are thus urgently needed. These predictions are hard to make due to non-linear responses and poorly understood feedback mechanisms between woody cover and other ecological responders, problems further amplified by the lack of long-term and large-scale datasets. We propose that a space-for-time analysis along an existing woody cover gradient overcomes some of these forecasting problems. Here we show, using an existing woody cover gradient (0–65%) across the Kruger National Park, South Africa, that increased woody cover is associated with (i) changed herbivore assemblage composition, (ii) reduced grass biomass, and (iii) reduced fire frequency. Furthermore, although increased woody cover is associated with reduced livestock production, we found indigenous herbivore biomass (excluding elephants) remains unchanged between 20–65% woody cover. This is due to a significant reorganization in the herbivore assemblage composition, mostly as a result of meso-grazers being substituted by browsers at increasing woody cover. Our results suggest that woody encroachment will have cascading consequences for Africa’s grazing systems, fire regimes and iconic wildlife. These effects will pose challenges and require adaptation of livelihoods and industries dependent on conditions currently prevailing.

## Introduction

Savanna is a unique biome since it covers a wide range of woody cover, yet neither “drifts” towards fully closed canopy forest/thicket, neither does it drift to fully open grassland—savannas maintain a spatially and/or temporally variable tree/grass mix under nearly all conditions of environmental forcing [1]. The explanation for this tree/grass co-existence has been the subject of many savanna studies and although the debate is still ongoing, it seems as if resource availability and variability (particularly rainfall), divergent rooting systems and disturbance regimes (like fire and herbivory) are some core conditions needed for co-existence [1,2,3]. This co-existence and variability in the tree/grass mix, often changing from open to dense over relatively small spatial (e.g., catena) or temporal (e.g., fire) scales, allows for a diversity of patterns, processes and functions to co-exist in a heterogeneous, sometimes shifting, tree/grass mosaic [4]. If a savanna with a heterogeneous tree/grass patchwork becomes significantly and uni-directionally either more open or more closed across multiple scales, then dominance shifts in species may result across many taxa and specialist species that prefer to occur on patches in the extremes (i.e., very open or very dense) may not persist. Also ecosystem services provided by savannas change depending on the tree/grass mixture—for instance, livestock grazing systems are dependent on the grass component and usually prefer more open systems [5,6], whereas the woody component is in some cases the exclusive energy source (cooking and heating) for many rural communities [7]. As such, a directional shift in woody cover towards a more homogenously open or dense state will have cascading effects for biodiversity, including humans, who depend on savanna heterogeneity. It has also been argued that savanna heterogeneity allows for increased resilience, productivity and coexistence of herbivores [8,9]. Woody encroachment, which we define as a unidirectional and a hard-to-reverse increase in woody cover, is thus a serious threat to the persistence of the species richness and functioning associated with structurally heterogeneous savannas.

Woody densification and encroachment occur world-wide and is a common phenomena in savannas [10]. Woody encroachment is a serious concern and could, stated rather dramatically, lead to the imminent “death of savannas” as some savannas may already be at a tipping point [11]. Various land-use practises like over-grazing and fire suppression have been proposed to explain this phenomena [1,12,13], and in recent years increased atmospheric carbon dioxide has been implicated as a global driver thereof [14,15,16]. The last 150 years has seen the near-linear increase of atmospheric carbon dioxide (CO_2_), with the current concentration of 390 ppm [17,18] higher than experienced by the biota on the planet for over 650,000 years [19]. It is predicted that this is resulting in a balance shift from grass to tree dominance in many savanna systems [15,20,21]. In short, woody encroachment, fuelled by both local and global drivers, is increasingly observed across savannas worldwide, and it is predicted that this process will continue and intensify as the atmospheric CO_2_ concentration keeps rising [22].

Although studies documenting shifts in the tree/grass ratio at various spatial and temporal scales are numerous, and our understanding is growing on the mechanisms driving these shifts [2,10,20,23], studies describing the cascading effects of the changing tree/grass balance are limited and mostly based on small-scale and controlled experiments comparing extreme scenarios (i.e., typically comparing open vs. dense scenarios, e.g., [24]). Instead, what is really needed to guide monitoring and management efforts is an understanding of how savanna systems may respond to shifts in woody cover (i) along a woody cover continuum (rather than only considering the extremes) and (ii) at ecosystem relevant scales (as opposed to small-scale experimental sites). Ecosystem-wide studies across a woody continuum represent more realistic scenarios and are more insightful as they reveal non-linearity and possible feedbacks [1,25] as well as scale dependent responses better than small-scale studies. We consider that the “space-for-time” approach we follow in this study along a woody cover gradient overcomes some of the shortcomings (see details later) of small-scale studies.

Managers and scientists will have to start making “best bet” predictions as to what the responses of systems might be due to environmental changes like woody encroachment [21]—the “needing-further-data” approach is paralyzing, setting mankind up to be unprepared and largely reactive in future. As such, Gordon & Prins [21] provided some “best bet” predictions on how herbivore species community trends may change due to increased woody cover under predicted future conditions. They hypothesized that an increase in the woody component will favour browsers due to increased browse availability. Furthermore, they predicted that increased woody cover will reduce grass biomass due to resource competition between the woody and herbaceous layers, which will subsequently reduce food available for grazers [21] (contrastingly, it has also been proposed that woody cover may facilitate the herbaceous layer, especially in very arid systems; [26,27]). However, because of multi-scaled responses, non-linearity and feedbacks in the system (e.g., [1,25]), it will be hard to test these predictions on whether and how grazer/browser systems may respond to shifts in the tree/grass mix [21]. In this paper we set out to contribute towards filling that knowledge gap by empirically testing some of these predictions. We achieved this by using various spatially explicit datasets which have recently become available for the 20,000 km^2^ KNP. We employed a “space-for-time” approach in which we compared fire return period, herbaceous biomass, herbivore abundance (separately for each of 12 herbivore species) and herbivore biomass (for all herbivore species combined) along a woody cover gradient within two contrasting geological settings. The assumption was that characterizing current patterns across a gradient of existing woody cover conditions (0–65%), with all other conditions kept similar, would allow making predictions as to how the system would respond in future to expected shifts in woody cover to a more dense system (or, elsewhere, to a more open system where that may be predicted). Our approach is analogous to studies exploring changes and trends along an altitudinal and/or rainfall gradient in order to predict how changes in rainfall and/or temperature may influence species distribution and/or migration/movement patterns (e.g., [28]).

## Materials and Methods

### Study site

The study was conducted in Kruger National Park (KNP), South Africa, which was established as a conservation area in 1902 and proclaimed a national park in 1926. The park covers about 20 000 km^2^ of semi-arid savanna with the full complement of large mammals indigenous to these African savannas. The rainfall ranges from 350 mm yr^-1^ in the north to 750 mm yr^-1^ in the south [29]. The area is predominantly underlain by soils of basaltic and granitic origin in the east and west, respectively.

### Datasets

Most of the data used in this project were collected in-house by the managing authority of the Kruger National Park (i.e. South African National Parks) with the necessary internal approval. Data collected by external researchers were registered and approved by SANParks Scientific Services and Conservation Management and had the necessary permissions and permits for conducting the fieldwork.

#### Geology data

Geological maps were used to delineate the park into different geological landscapes [29]. Granite and their erosion products dominate the west of the park (about 9000 km^2^), while the eastern sector is predominantly underlain by basalt-derived erosion products (about 5500 km^2^). The clayey basaltic soils are generally more nutrient rich than the sandy granitic soils. The geology of the remainder of the park include rhyolite, gabbro, ecca shale and aeoleon sands (in order of prevalence), but these cover too small areas with too few data points spread across the woody cover gradient to allow for separate analysis.

#### Woody cover data

The woody cover map was generated by calibrating 73 field points (collected in 2006) with four Landsat ETM+ scenes (collected in 2000 and 2001) and eleven JERS-1 Synthetic Aperture Radar (SAR) scenes (collected in 1995–1996). The field plots (250m x 250m) were laid out to cover the rainfall gradient and main geology of the park and to represent the range of woody cover existing in the park. At each plot woody canopy cover (i.e. fraction of skylight orthogonal to the surface obstructed by plant canopies at least 1.3m tall) was estimated using a hand-held spherical densitometer. The cover for each plot was estimated by averaging 30 measurements collected on a regular grid. The plot measurements were then used to calibrate a multiple regression model between the remote sensing data (including spectral bands and indices derived from the imagery) (predictor variables), and the field collected woody cover (response variable). The best predictive model was selected from the models containing all the possible variable combinations using the Akaike information criterion (AIC). The best model was then used to extrapolate woody cover across the entire park at a 90m resolution using the remote sensing variables. The model predictions were validated using an independent Light Detection and Ranging (LiDAR) dataset obtained from the Carnegie Airborne Observatory in 2008 (see [30] for details on the LiDAR system). For this study we averaged the 90m woody cover map to a 1km resolution to be compatible with the other datasets used in this study. This woody cover map is described in [31] and has been successfully used in habitat suitability modelling [32].

#### Average herbaceous biomass data

We used a 1 km^2^ co-kriged interpolated surface of average herbaceous biomass. This interpolation employed average long-term field-collected disc-pasture meter herbaceous biomass as primary variable and vegetation indices derived from satellite imagery as secondary variable. Herbaceous biomass was measured each year between 1989–2005 at more than 500 plots spread throughout the park. Plots were 50m x 60m and 100 disc pasture meter measurements were collected on transects within each plot. Disc-pasture meters estimate herbaceous biomass by measuring the height a disc of standardised dimensions is obstructed from the ground (by the herbaceous layer) when dropped from a fixed height (i.e. the height relates to the herbaceous biomass under the disc). The 100 height measurements at each plot were then averaged before using a calibration equation specific to Kruger National Park to convert height (cm) into herbaceous biomass (kg.ha^-1^) [33]. The annual herbaceous biomass at each plot was then averaged over the entire period to estimate the plot’s long-term herbaceous biomass. These long-term plot-based herbaceous biomass estimates were thereafter interpolated across the entire park by co-kriging, using a time-series of 1km^2^ NDVI metrics derived from AVHRR imagery (employing 19 673 grid cells). This co-kriged herbaceous biomass surface is described in more detail in [34] and has been successfully used in habitat suitability modelling [32,34] and fire behaviour studies [35].

#### Fire return period data

KNP has kept extensive records of fire scars, resulting in a spatially explicit fire scar history since 1941 (see [36] for how these fire scar maps have been created as technology changed from hand-drawn maps historically to satellite derived fire scars currently). We used the average fire return period measured over 67 years (between 1941 and 2006) (described in [35]).

#### Herbivore density, biomass and diversity

We used aerial census data collected in the dry-season (May-August) between 1987–1993 to estimate long-term average herbivore density, biomass and diversity. The aerial census data consists of herbivore counts mapped from a fixed-wing aircraft flying regularly spaced strip transects covering the park from north to south. The aerial census dataset is described in detail in [37]. Although aerial surveys have been conducted in the KNP for many years, we used the 1987–1993 subset as it represents the period of (i) the highest spatial resolution of the surveys, (ii) the most complete yearly coverage, and (iii) the most consistent methodology between years. The far north of the park was not surveyed regularly and was therefore omitted from the analysis pertaining to herbivores. However, since the far north of the park did not contain any granitic derived landscapes, aerial census data was available across the entire granitic-derived landscapes of KNP, whilst a small proportion of the basaltic-derived landscapes had to be excluded due to incomplete aerial census data on the far northern basalts.

For twelve herbivore species the survey data were of high quality and are included in the analysis (Table 1). Due to elephant’s unique diet, including bark, roots, twigs and branches (in addition to grass and browse more commonly utilized by other herbivores) this species was excluded from the analysis. Furthermore, more than 2600 elephants were culled or translocated during the study period [38] and their number has continued to grow rapidly since culling ceased in 1994, suggesting that this species is still not at a stage of natural regulation and fluctuation and as such may confound patterns based on species that are at “naturally regulated” levels and that has not been intensively managed in recent years.

**Table 1:** Average adult body mass of herbivore species included in analysis (body mass from [56,57]) (arranged from smallest to largest).

Species	Body mass (kg)	Feeding guild
Impala	45	Mixed feeder
Tsessebe	133	Grazer
Kudu	140	Browser
Blue Wildebeest	180	Grazer
Waterbuck	205	Grazer
Sable	230	Grazer
Zebra	260	Grazer
Roan	270	Grazer
Eland	460	Mixed feeder
Buffalo	520	Grazer
Giraffe	830	Browser
White rhino	1727	Grazer

Three herbivore variables were derived from the aerial survey data explained above. Firstly, average herbivore density was calculated by averaging aerial herbivore counts observed between 1987–1993 to estimate the average herbivore density (ind.km^-2^) for each species. Secondly, Total Herbivore Biomass (THB) (kg.km^-2^) was estimated by adding the biomass of all the herbivore species occurring in the same grid cell, and was calculated as follows (for each 1 km^2^ grid cell):

$$THB=\sum_{{}_{i}}^{n}Di*Mi$$ (1)

where

Di = average density of species *i* over the seven years,

M*i* = typical body mass of species *i* (see Table 1), and

n = 12 (number of herbivore species).

Thirdly, based on the average abundance of the 12 herbivore species listed in Table 1, the Shannon-Wiener diversity index was calculated for each 1 km^2^ pixel.

### Data analysis

All datasets described above were re-sampled to a common grid of 1 km^2^ in order to do regression analysis between the series of overlaid grids. As such, the woody cover grid was linearly regressed with the three herbivore variable grids outlined in the previous section (i.e. average herbivore density, total herbivore biomass, and herbivore diversity index) to determine how these variables change along a woody cover gradient. The relationships between woody cover and all the variables were also visually presented as line graphs by averaging the parameters in woody cover bins of 5% increments. All spatial analyses were conducted in ArcMap (ver. 9.2) and its extensions and statistical analysis in Statistica (version 11).

## Results

**Fig 1:**
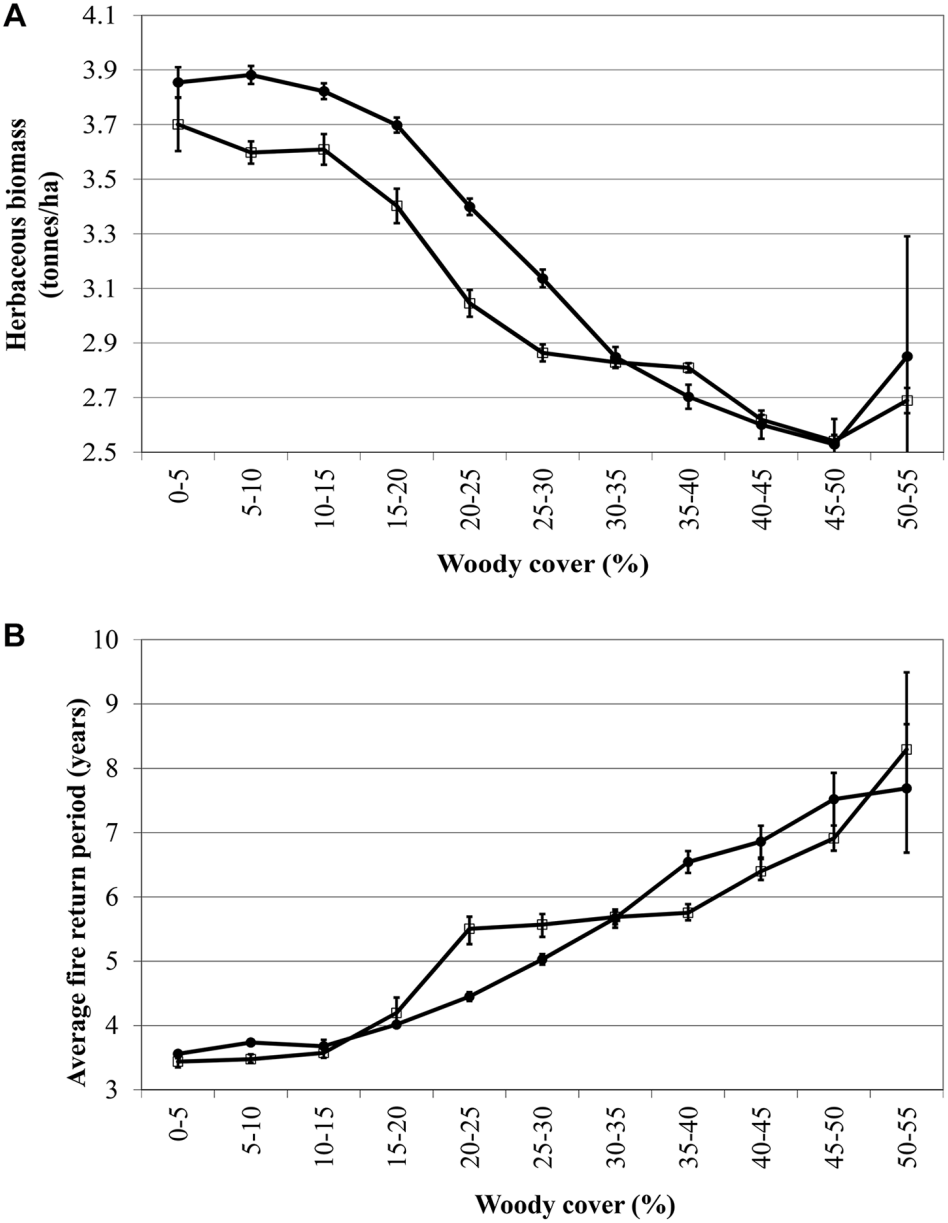
Average herbaceous biomass (ton.ha^-1^) and fire return interval (years) observed along a woody cover gradient. (basalts: solid circles; granites: open squares) (standard error bars included). Average long-term herbaceous biomass decrease (a), whilst average long-term fire return period increase (b) along a gradient of increasing woody cover.

Herbaceous biomass decreases and fire return period increases with increasing woody cover (Fig 1). These patterns were observed in both geological landscapes studied.

**Fig 2:**
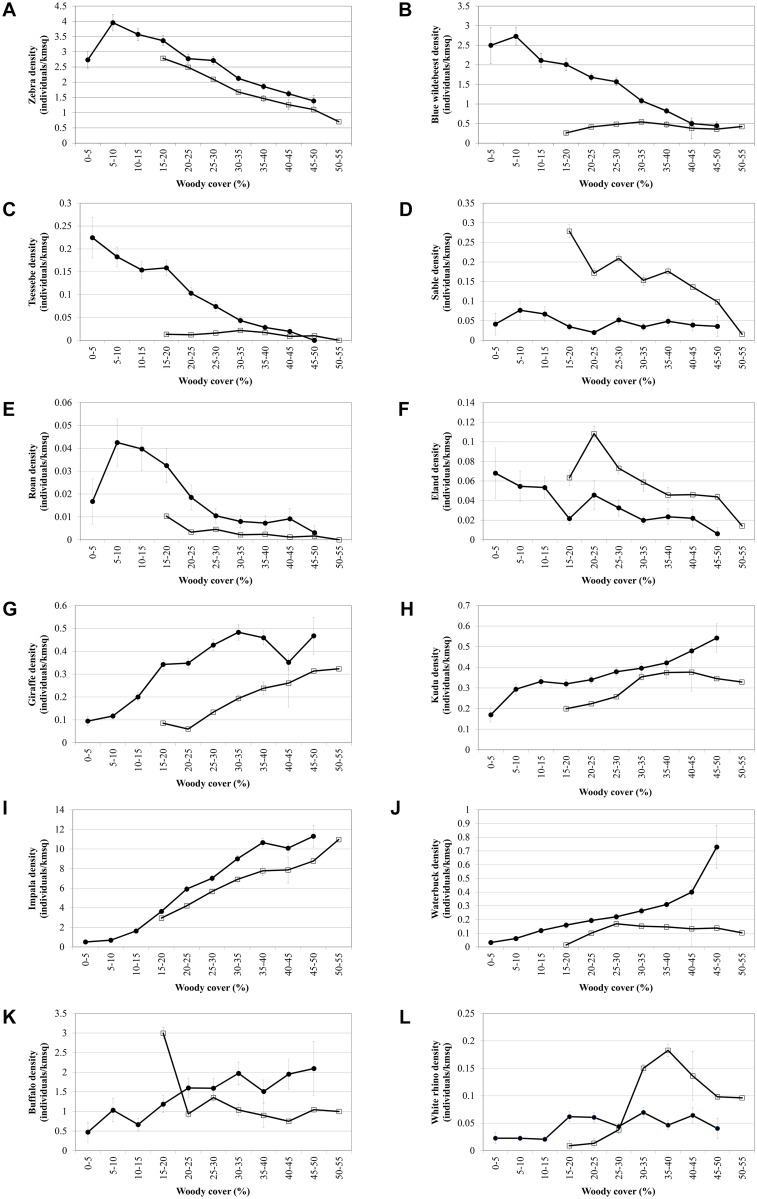
Average herbivore density (individuals.km^-2^) observed along a woody cover gradient. (basalts: solid circles; granites: open squares) (standard error bars included). Meso-grazers including (a) zebra, (b) blue wildebeest, (c) tsessebe, (d) sable and (e) roan, as well as the mixed feeding (f) eland decrease along a gradient of increasing woody cover. Browsers including (g) giraffe and (h) kudu, as well as the mixed feeding (i) impala increase along the woody cover gradient. Some species did not show a consistent trend across the geologies, including (j) waterbuck, (k) buffalo and (l) white rhino (refer to Table 2 for the slope coefficients).

The five meso-grazer species (plains zebra–*Equus burchellii*, blue wildebeest–*Connochaetus taurinus*, sable antelope–*Hippotragus niger*, roan antelope–*H*. *equinus*, tsessebe–*Damaliscus lunatus*) as well as one of the mixed feeding species (common eland–*Tragelaphus oryx*) decrease along the woody cover gradient, whilst the two browsing species (greater kudu–*Tragelaphus strepsiceros*, and giraffe–*Giraffa cameleopardalis*) and a mixed feeding species that mostly browse in the dry season (impala–*Aepyceros melampus*) [39] increase with increasing woody cover (Table 2; Fig 2). (Note: Because sable is virtually absent from basalts (0.044 ind.km^-2^) compared to granites (0.150 ind.km^-2^), we focused on the trend on the granites as a more reliable indicator of sable’s tendency to decrease with increasing woody cover). Buffalo (*Syncerus caffer)*, white rhinoceros (*Ceratotherium simum*) and waterbuck (*Kobus ellipsiprymnus)* showed no consistent trends along the woody gradient on the two geological landscapes (Table 2; Fig 2).

**Table 2:** Slope coefficients of herbivore density (individuals.km^-2^) along a gradient of increasing woody cover (e.g. for a 10% increase in woody cover on the basalts, the impala density increase by 3 individuals.km^-2^ whilst the zebra density decrease by 0.6 individuals.km^-2^). This illustrates that browsers increase with increasing woody cover, whilst most grazers decrease. (**** 0.01% significance level; *** 0.1% significance level; ** 1% significance level; * 5% significance level; when applying a Bonferroni correction for 24 tests, only species indicated as significant at the ≤0.1% level will remain significant at the 5% level).

Species	Basaltic	Granitic
***Species increasing along a gradient of increasing woody cover (i*.*e*. *positive slope coefficients)***		
Impala	0.308 ****	0.178 ****
Kudu	0.005 ****	0.004 ****
Giraffe	0.009 ****	0.008 ****
***Species decreasing along a gradient of increasing woody cover (i*.*e*. *negative slope coefficients)***		
Zebra	-0.063 ****	-0.050 ****
Blue wildebeest	-0.057 ****	-0.005 **
Tsessebe	-0.005 ****	-0.001 ****
Sable	-0.001 NS	-0.006 ****
Roan	-0.001 ****	-0.0001 *
Eland	-0.001 **	-0.002 ****
***Species showing inconsistent responses along a gradient of increasing woody cover across the geologies***		
Waterbuck	0.009 ****	-0.00003 NS
Buffalo	0.003 ****	-0.021 **
White rhino	0.001 ****	NA #

^#^ Not appropriate to calculate slope coefficient for white rhino on granites due to non-linear relationship (see Fig 2).

**Fig 3:**
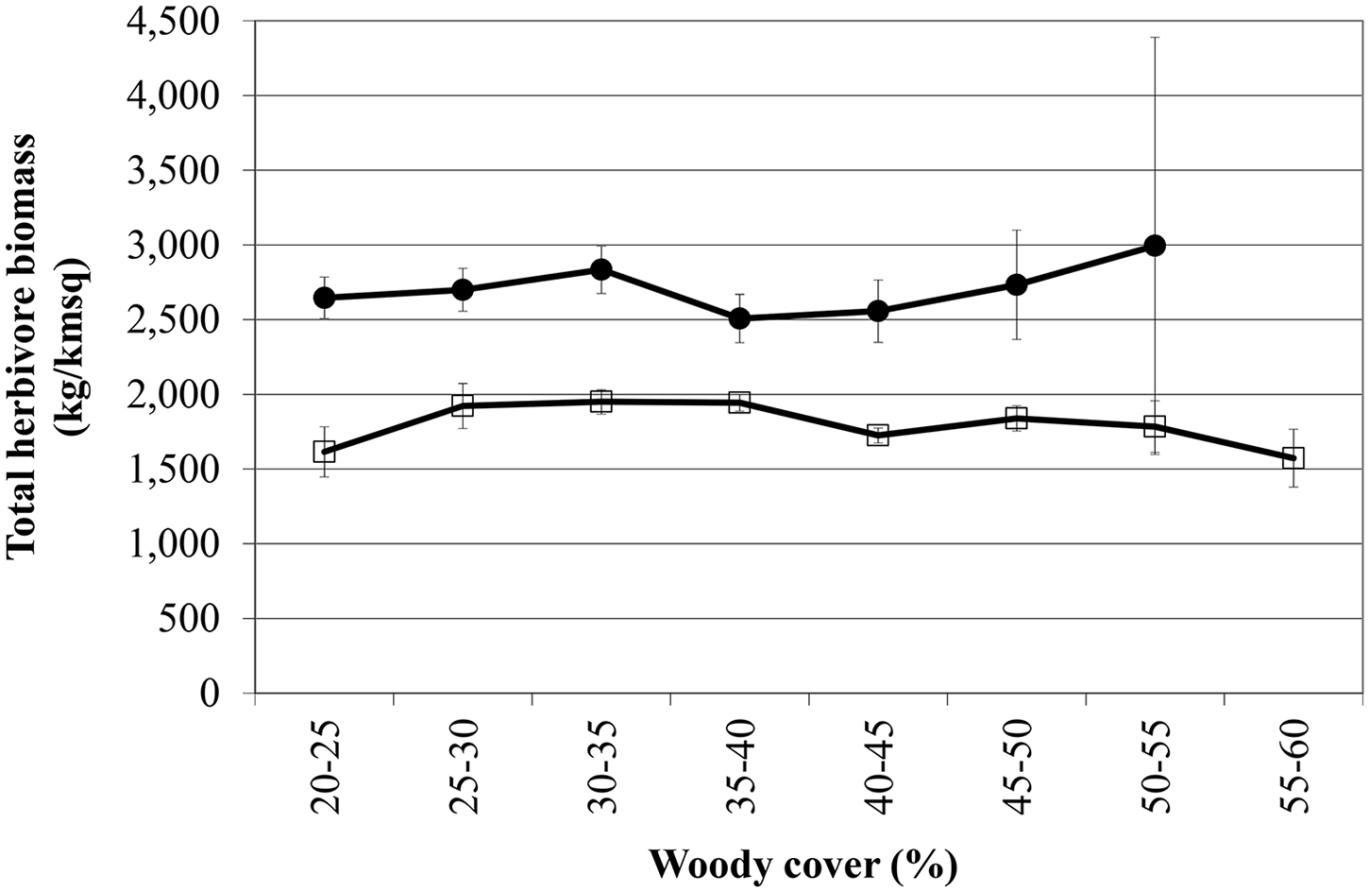
Average Total Herbivore Biomass (THB) (kg.km^-2^) observed for 12 large herbivore species along a woody cover gradient. (basalts: solid circles; granites: open squares) (standard error bars included). As expected the nutrient-rich basalts support higher biomass than the nutrient-poor granites. Surprisingly, even though nine of the 12 individual species studied show significant directional increases or decreases along the woody cover gradient (see Fig 2 and Table 2), when all species are combined the average THB stays constant between 20–65% woody cover with no change in productivity (refer to Table 3 for slope coefficients).

**Fig 4:**
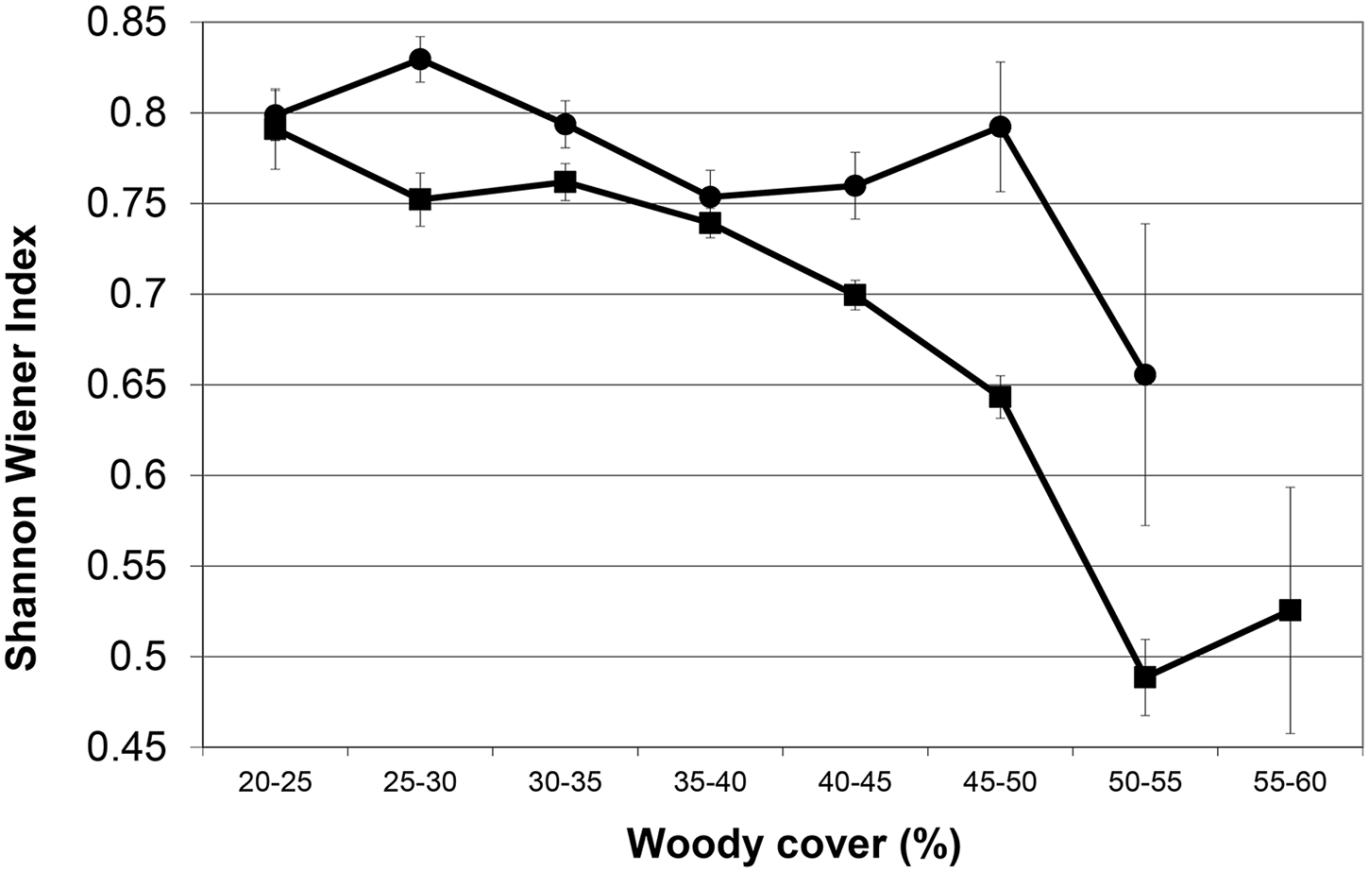
Shannon Wiener Diversity Index along a woody cover gradient. (basalts: circles; granites: squares) (standard error bars included). Note how with increasing woody cover the diversity index decrease, with highly significantly negative slope coefficients (basalt: -0.0025 (p = 0.005; n = 3570); granite: -0.0078 (p<0.0001; n = 8682)).

**Fig 5:**
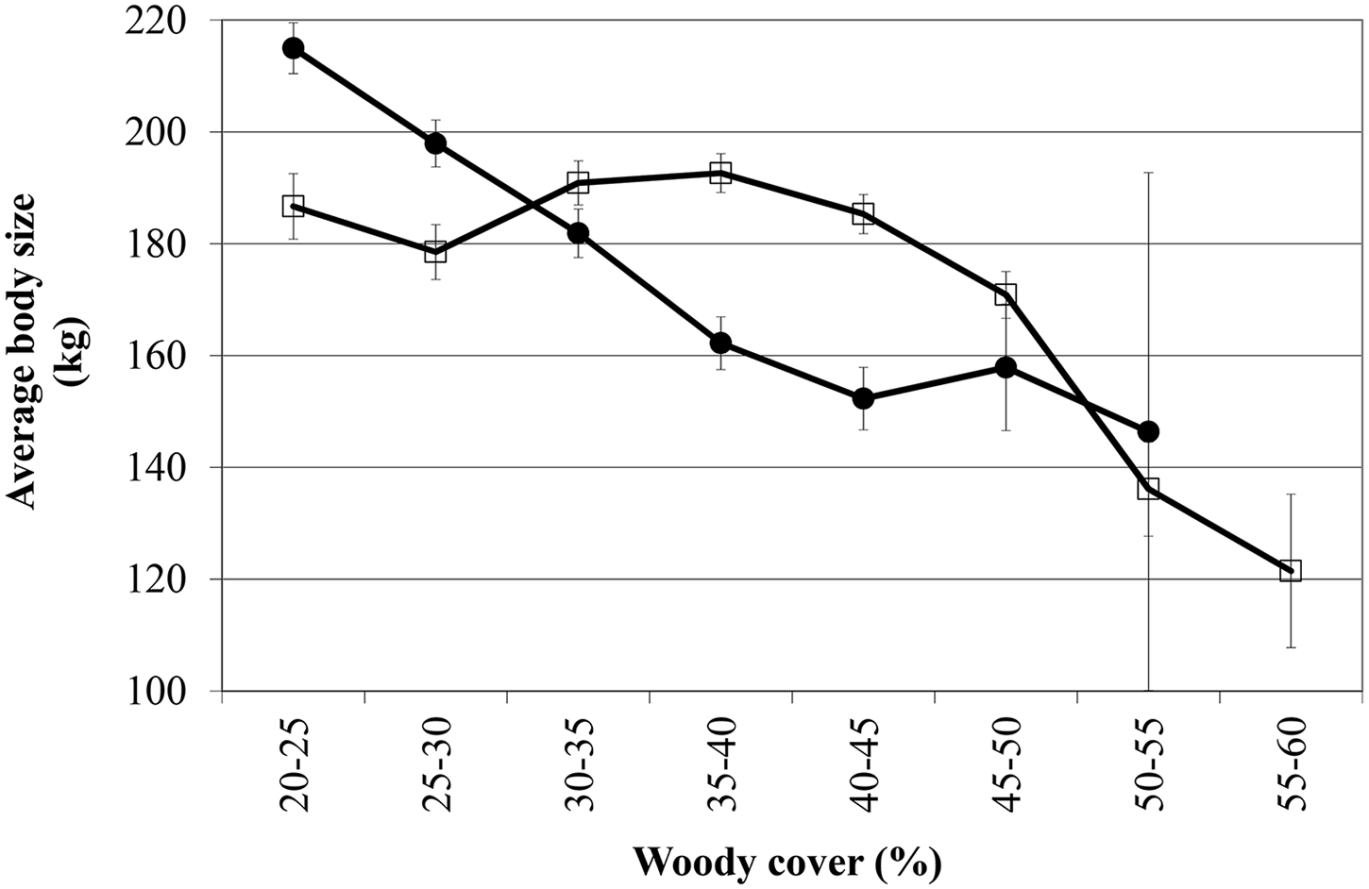
Average body size of herbivores along a woody cover gradient. (basalts: solid circles; granites: open squares) (standard error bars included). Note how with increasing woody cover the average body size decrease, with negative slope coefficients (basalt: -2.94071 (p<0.0001; n = 3570); granite: -1.20524 (p<0.0001; n = 8682)).

When all species are combined the average Total Herbivore Biomass (THB) remains constant between 20–65% woody cover in both geological landscapes (THB: Fig 3 & Table 3). As species respond differently to the woody gradient, the Shannon Diversity Index decreases significantly (Fig 4), as does the average body size of herbivores (Fig 5).

**Table 3:** Slope coefficients of average Total Herbivore Biomass (THB) (kg.km^-2^) along an increasing woody cover gradient (20–65%) (**** 0.01% significance level; *** 0.1% significance level; ** 1% significance level; * 5% significance level; NS non-significant). The slope coefficients were calculated for all species combined (column 3), as well as for subsets of species based on whether they increase (column 4), decrease (column 5) or have variable responses (column 6) along a gradient of increasing woody cover. Note how the slopes are not significantly different from zero when considering all species combined (column 3), as opposed to highly significant positive (column 4) and negative (column 5) slopes for the suits of species increasing and decreasing respectively along a gradient of increasing woody cover. This illustrates that although herbivore biomass will get partitioned differently between species with increasing woody cover, the total herbivore biomass when considered across all species will remain constant between 20–65% woody cover.

Geology	Sample size (n)	THB of all species	THB of species increasing with rising woody cover (impala+giraffe+ kudu)	THB of species decreasing with rising woody cover (zebra+blue wildebeest+tsessebe+ sable+roan+eland)	THB of species showing variable responses with rising woody cover (buffalo, waterbuck & white rhino)
Basalt	3570	-4.328 NS	15.325 ****	-28.763 ****	9.110 NS
Granite	8682	-8.757 NS	15.101 ****	-17.215 ****	-6.642 NS

## Discussion

As hypothesized, browser densities increase as woody cover increases, whilst most meso-grazer densities decrease along the same gradient. Our results also clearly illustrates that this is most probably due to changed forage availability with herbaceous biomass decreasing as woody cover increases. To our knowledge these patterns have not been demonstrated previously over a woody cover gradient spanning most of the dynamic range typical of savannas (0–65%) (cf. [2]) or based on such a large suite of species and at an ecosystem-scale. We postulate that shade and water dependence of the large-bodied and thermally-challenged buffalo (*Syncerus caffer)* and white rhinoceros (*Ceratotherium simum*), as well as the strong water association of waterbuck (*Kobus ellipsiprymnus*) [40] introduce trade-offs with forage availability (cf. [34]). These trade-offs may confound the relationship that is observed for the other grazer species: as such these three species show no consistent trends along the woody gradient on the two geological landscapes. For example, on the granites waterbuck and buffalo respectively showed no trend and a negative trend as woody cover increases, yet an increasing trend is observed on the basalts. We postulate that this may be due to the fact that there is a gradient of increasing woody cover closer to rivers on the basalts as opposed to the granites (see Fig 5 in [35]), and as such the water dependence of these two species overrides the importance of the woody gradient. It is also possible that these three species select habitat at a different scale than the scale that was considered in this study (1km).

Our most unexpected, and in our opinion most insightful finding, was that even though we observed statistically significant directional increases or decreases along the woody cover gradient for most of the individual herbivore species (as described above), these species balance each other out, almost “kilogram for kilogram”, along the woody cover gradient. This is even more remarkable considering the wide range of average body mass between the species (ranging from the 45kg impala to the 1700kg white rhino; Table 1). A similar constancy in herbivore biomass, but in time and not in space as the case here, was observed in East Africa where individual species fluctuated quite widely in their numbers but where the species assemblage as a whole showed constancy in mass over time [41]. However, even though the herbivore productivity and metabolic requirements do not change along a 20–65% woody cover gradient, the Shannon-Wiener Diversity Index decreases along the gradient in both geological landscapes as a result of the assemblage changes (Fig 4). This decreasing diversity is not that surprising considering the fact that there are more grazing than browsing large bodied herbivore species in Africa. Therefore, whilst biomass of the herbivore assemblage do not change with increasing woody cover, the change in woody cover causes a drastic restructuring of the herbivore assemblage as can be seen from the individual species responses as well as the community-measured Shannon diversity index. In agricultural scenarios woody encroachment usually reduces biomass production [42,43]. In a recent large-scale study in the north and south Americas, it was reported for each 1% increase in woody cover a reduction of between 0.6 and 1.6 reproductive cows per square kilometer [6]. However, when considering a natural, multi-species herbivore assemblage, our results suggest that when the system gets reorganized in terms of herbivore species composition, biomass production remains constant (as expected, the biomass production is higher on the nutrient-rich basalt soils as opposed to the nutrient-poor granitic soils) (Fig 3). As such, our results imply that the viability of game farming/ranching as a land-use for producing protein will not change considerably under woody encroachment although the biomass will be “packaged” differently (this result seems to hold up to at least 65% woody cover as the upper range of our study). However, even though herbivore biomass production may remain viable, tourism may be negatively affected as visitors have indicated that even if animal numbers stay the same, but they see fewer animals due to reduced visibility brought about by woody encroachment, they will be dissatisfied and may reconsider their visit to a particular park [44]. Furthermore, even though productivity of herbivore biomass may be maintained under woody encroached conditions, we postulate that this “repackaging” of herbivore biomass may have other unexpected cascading effects as well. For example, as a result of the herbivore assemblage re-structuring, the average body size of herbivores decrease significantly along the woody cover gradient (Fig 5). The change in prey species prevalence and average prey species size, as described above, may therefore potentially influence the competitiveness of different predator species [45] and/or influence the optimal group-size and hunting behavior of social predators [46]. For example, it has been shown that blue wildebeest is one of the favorite prey species of lion, which is the apex predator in Kruger, during wet periods [47]. Here we show that blue wildebeest will decline under encroached conditions, which will lead to lions switching to other prey species, which may in turn require a change in their hunting strategy and/or group size and composition (see, e.g., [48], indicating that male lions prefer denser areas to hunt as opposed to female lions that are more successful in open areas).

Besides the herbivore responses predicted for changing woody cover, our results also clearly illustrate that increased cover will reduce herbaceous biomass and reduce fires (i.e., decrease fire frequency and increase fire return interval; Fig 1). Although we measured the patterns and not the underlying mechanisms, we postulate that increased woody cover causes reduced herbaceous biomass due to resource competition. The reduced herbaceous biomass (which is the dominant fuel load in savanna fires) in turn result in longer inter-fire periods and fires of lower intensity, most likely inducing a reinforcing feedback loop [1]. As such one can predict that with increasing woody encroachment, as either caused by increased atmospheric CO_2_ levels or poor landuse practises, fire intervals will increase, thus further stimulating woody encroachment, even in extreme cases resulting in the conversion of mesic savanna to closed-canopy forest or thicket [14]. Furthermore, a reduction of fire frequency and intensity will not only have effects on biodiversity [49,50], but may also have significant influences on global atmospheric fluxes. The annual flux of particulate carbon into the atmosphere from vegetation biomass burning in African savannas is in the order of 8 Tg C, which rivals particulate carbon emissions from anthropogenic activities in temperate regions [51]. With more woody vegetation, less burnable grass and fires fewer and longer apart, emissions from savanna fires to the atmosphere will change, impacting on the global carbon cycle.

In summary, our results predict that if woody densification continues due to inappropriate landuse practises and increasing atmospheric CO_2_, Africa will become less grassy, will burn less frequently and grazers like zebra and wildebeest will be replaced by browsers like giraffe and kudu and by mixed-feeding impala, possibly with cascading effects for the predator guild. As support for our space-for-time interpretation, we note that species preferring more woodier conditions (kudu, giraffe and impala) are at, or close to, all-time highs since population estimates started in the 1960s in Kruger National Park, yet species preferring more open landscapes (blue wildebeest, sable, roan, tsessebe and eland) are at or close to all-time low population estimates (zebra is an exception, but it may be due to its different gut-morphology, being a hind-gut fermentor rather than a ruminant). Various causes, including artificial water provision, predation [52] and climate-change induced forage quality deterioration [53] have been postulated for these changes in the herbivore assemblage, but we propose that a thickening woody layer (possibly caused by increased CO_2_ since the 1950s) may be another explanatory factor, possibly interacting with some of these other system changes, to be considered.

Our study has some limitations and assumptions. Firstly, we assume that one can infer from a prevailing woody cover gradient how certain patterns and processes will change under future woody thickening conditions. Although we believe this assumption is relatively robust and intuitive, we acknowledge that it may not fully hold under all conditions and for all variables. For example, woody thickening is often associated with one or a few woody species increasing disproportionately in density in an area (e.g. sickle bush *Dichrostachys cinerea*), and as such an area exposed to woody densification may have a different composition and vertical structure compared to an area of similar woody cover where cover is naturally high and not due to the process of woody densification. A second potential limitation of our study is the time stamps of our datasets that are variable. However, we do not consider this a major concern, as there is no indication that the long-term fire return period, herbaceous biomass and herbivore distribution patterns have changed considerably (especially in relative terms) over the study period (e.g. the areas that respectively burn frequently and rarely have not changed; [35]). Also, at the relatively coarse scale and large extend at which we conducted our analysis, localised changes over time will not pose a problem. Finally, we have justified our decision to exclude elephants in the methods section, however, we realise that by doing so we exclude an important component of herbivore biomass in African savannas. When elephant biomass is included in the analyses, an increasing trend of biomass against woody cover is observed instead of the currently reported slope of zero in Fig 3. It is unclear at this stage whether this result is influenced by the fact that Kruger’s elephants have been highly managed in the past which may have density and spatial legacy effects, and/or due to elephant’s atypical diet. As such, we don’t know how this pattern may change after many years of non-active management. Elephants can make up more than 50% of large herbivore biomass, and are a critical component of biomass in African savannas [54]. We therefore acknowledge that Fig 3 may indeed look different when elephants are included, and this could be an important focus area for future studies.

## Conclusions

Our results provide empirical support for various hypotheses that were previously posed, but that largely remained untested at large spatial scales. As such, our results predict that the increases in woody cover observed and forecasted for African savannas may have wide-ranging impact on various patterns and processes at the regional scale (e.g., changing herbivore assemblage structure) as well as the global scale (e.g., changed carbon emissions from wild fires). These changes may have cascading effects on atmospheric cycles, biodiversity, tourism, the livestock industry and subsistence livelihoods, and it will require novel ideas to counter or adjust to these changes (for instance, the application of high intensity “firestorms” under conditions normally deemed inappropriate for setting management fires in order to combat woody encroachment) [55]. Considered positively, our results illustrate the ability of savanna ecosystems to possibly adapt (through changing herbivore composition) to changing woody cover and to maintain productivity (by maintaining a constant herbivore biomass), as opposed to livestock farming where increased woody cover usually results in reduced biomass productivity [6,42,43]. As such, our results highlight important challenges, but also possible solutions and opportunities, for commercial industries like meat producing agriculture, hunting and tourism as well as subsistence livelihoods that are dependent on the productivity of wild herbivores and/or livestock grazing.
